# Knowledge, attitude, and practices of households toward tuberculosis transmission and early diagnosis

**DOI:** 10.1038/s41598-026-48332-6

**Published:** 2026-04-20

**Authors:** Abdulkadir Ibrahim Adow, Shaadiya Mohamed Ali, Bashir Mohamed Abdi, Sharmake Gaiye Bashir

**Affiliations:** 1 Faculty of Health Sciences and Tropical Medicine, Somali National University, Mogadishu, Somalia; 2 Center for Health Research and Innovation, Somali National University, Mogadishu, Somalia; 3 Faculty of Health Science, Salaam University, mogadishu, Somalia

**Keywords:** Tuberculosis, Knowledge, Attitudes, Practices, Household, Early diagnosis

## Abstract

Tuberculosis (TB) remains a major public health challenge, particularly in fragile, high-burden settings like Somalia. Household knowledge, attitudes, and practices (KAP) critically influence early diagnosis, treatment adherence, and community transmission. This study assessed KAP toward TB transmission and early diagnosis among households in Mogadishu. A community-based cross-sectional study was conducted from December 2025 to February 2026 among 403 adult household representatives in Mogadishu. Multistage and systematic random sampling were employed. Data were collected using a structured interviewer-administered questionnaire and analysed with Stata 17. Composite scores for knowledge, attitude, and practice were calculated (range 0–5), and associations with sociodemographic factors were examined using t-tests and ANOVA. Most participants were aware of TB (85.4%), recognized airborne transmission (87.1%) and persistent cough as a key symptom (86.4%), and knew it is curable if diagnosed early (83.1%). Only 48.6% correctly identified TB as caused by bacteria. Positive attitudes were common, with 88.6% perceiving TB as serious and 83.1% believing in completing treatment; however, only 50.6% felt comfortable interacting with TB patients. Mean KAP scores were moderate to good: knowledge 3.91 ± 1.23, attitude 3.92 ± 1.28, and practice 3.92 ± 1.15. KAP scores varied significantly by age, sex, marital status, education, and household size. Households in Mogadishu demonstrate general awareness of TB but substantial gaps remain in biomedical knowledge and zoonotic risk understanding, alongside persistent stigma. Culturally tailored, community-based interventions targeting misconceptions, promoting early diagnosis, and strengthening household engagement are critical for effective TB control.

## Introduction

Tuberculosis (TB) remains a profound global public health challenge, persisting as the leading cause of death from a single infectious agent worldwide^1–3^, Caused primarily by the bacterium Mycobacterium tuberculosis, the disease is estimated to infect approximately one-quarter of the world’s population, with 10 million people falling ill and 1.6 million dying annually as of 2017^1,3,4^. The epidemiology of TB is inextricably linked to its airborne transmission dynamics, where infectious bacilli are expelled into the air through coughing, sneezing, or spitting, placing entire communities at risk^5–7^, The burden is further intensified by the emergence of multidrug-resistant TB (MDR-TB), a significant threat described as "Ebola with wings" due to its high transmission risk and the complexities associated with its treatment^8,9^. Despite the implementation of global strategies such as the "End TB Strategy," a substantial gap persists between incident and notified cases, largely driven by delayed diagnosis and treatment gaps that facilitate ongoing community transmission^10^. The public health burden of TB is disproportionately concentrated in low- and middle-income countries (LMICs), with Sub-Saharan Africa (SSA) accounting for approximately 25% of the global TB burden^11–13^. In this region, the epidemic is characterized by high rates of human immunodeficiency virus (HIV) co-infection, which serves as the most critical risk factor for the activation of latent TB into active disease^6,14,15^. Structural determinants such as widespread poverty, malnutrition, urban overcrowding, and substandard housing conditions create environments conducive to rapid transmission^1,16,17^. Furthermore, many SSA countries suffer from fragile health systems, limited diagnostic capacity, and significant socioeconomic barriers, including the high cost of seeking care and the social stigma that forces individuals to conceal their status^1,2,9,12^. The intersection of these factors leads to delayed presentation at health facilities, often occurring only when the disease has reached an advanced and highly infectious stage^11,17^.

Within this regional context, Somalia faces an exceptionally severe epidemiological situation, reporting some of the highest TB incidence rates in the world^1^. The disease is endemic and ranks as a leading cause of morbidity and mortality, with estimated incidence and prevalence rates of 274 and 513 per 100,000 population, respectively^1^. The collapse of the national TB Control Programme during the civil war created an unprecedented barrier to stability and effective disease management^1^. Currently, it is estimated that out of approximately 12,000 sputum-positive cases occurring annually, only one-third are detected and receive supervised treatment^1^. This case detection gap is exacerbated by fragile infrastructure, displacement, and extreme socioeconomic vulnerability, particularly among the 15–49 age group^1^, Traditional beliefs and a lack of awareness regarding the biological causes of TB frequently lead to reliance on informal health providers, further delaying early diagnosis and treatment initiation^1,17^. Knowledge, attitudes, and practices (KAP) serve as critical determinants of TB prevention and control because they directly influence health-seeking behaviors and treatment adherence^6,11,18^. Knowledge encompasses the ability to pursue and use information regarding etiology, symptoms, and transmission, while attitudes reflect the reactions and beliefs formed based on that knowledge^5,19^, Household awareness of transmission routes, such as the recognition that TB is airborne rather than hereditary or caused by superstitions, is fundamental for implementing protective measures^6,16,20^, Symptom recognition, particularly the identification of a persistent cough (chifuwa chachikulu), is a prerequisite for early diagnosis^6,17^. However, low risk perception and the pervasive stigma associated with TB and HIV often lead to the intentional concealment of symptoms, creating significant hurdles for effective contact tracing and case identification^12,17^. Existing global and regional evidence reveals significant inconsistencies in community KAP levels, reflecting a scarcity of context-specific data in fragile settings^1,2^. Studies in Ethiopia and South Africa have reported relatively high awareness levels, yet significant misconceptions regarding transmission through sharing utensils or kissing persist^15,16,21^. In contrast, research in settings like Gambia and Jordan identifies substantial gaps in knowledge about the costs of treatment and the zoonotic transmission pathways of bovine TB^3,10,11^. Methodological limitations in previous literature, such as a heavy focus on urban populations or patients already in care, often fail to capture the perspectives of healthy households or those in remote, conflict-affected areas^16,22–24^, Particularly in Somalia, there is a notable lack of robust household-level data that addresses how traditional beliefs and the aftermath of conflict shape current TB-related practices^1^.

Closing these knowledge gaps is essential for designing targeted health education interventions and strengthening community engagement^1,5,25^. Evidence from other regions suggests that tailored sensitization programs utilizing mass media, especially radio, and the engagement of community health workers can significantly improve early case detection and reduce stigma^12,16,25^. For Somalia, such evidence is vital for supporting national control strategies and integrating One Health approaches that address both human and bovine TB transmission^1^. Understanding the specific barriers within Somali households, from the fear of social isolation to misconceptions about curability, allows for the development of culturally appropriate communication pathways that can bypass the limitations of a weakened formal health system^1,17^.

The purpose of this study is to assess the knowledge, attitudes, and practices of households in Mogadishu, Somalia, toward TB transmission and early diagnosis.

## Methodology

### Study design and period

A household based cross-sectional study was conducted to assess knowledge, attitudes, and practices (KAP) of households toward tuberculosis (TB) transmission and early diagnosis. The study was carried out between December 2025 and February 2026.

### Study setting

The study was conducted in Mogadishu, Somalia, an urban setting characterized by high population density, internal displacement, overcrowding, and limited health infrastructure. These contextual factors contribute to ongoing TB transmission and delayed case detection. Public TB diagnostic and treatment services are delivered through facilities operating under the National Tuberculosis Control Program.

### Study population and eligibility criteria

The study population comprised adult household representatives residing in selected districts of Mogadishu. A household was defined as individuals living together under one roof and sharing meals. Eligible participants were adults aged 18 years and above who had lived in the area for at least 6 months. In households with multiple eligible adults, one respondent was selected. Individuals who were critically ill or unable to provide informed responses were excluded.

### Study variables

The primary outcome variables were composite knowledge, attitude, and practice scores related to TB transmission and early diagnosis.

Knowledge was assessed using five items addressing:

- Awareness of TB,
- Understanding that TB is caused by germs/bacteria,
- Recognition of airborne transmission via coughing/sneezing,
- Identification of persistent cough as a key symptom, and
- Awareness that TB is curable if diagnosed early.

Attitude was measured using five statements evaluating perceptions of TB severity, early health-seeking Behavior, willingness to encourage care, comfort interacting with TB patients, and belief in completing the full course of treatment.

Practice was assessed using five items reflecting reported or intended health-seeking behaviours related to TB symptoms and treatment adherence.

Each correct or favourable response was scored as 1 and incorrect/unfavourable responses as 0. Composite scores ranged from 0 to 5 for each domain, with higher scores indicating better knowledge, more positive attitudes, or appropriate practices.

Independent variables included age, sex, marital status, education level, and household size.

### Sample size determination

The sample size was calculated using the single population proportion formula:

$${n}_{0}=\frac{{Z}^{2}\times p(1-p)}{{d}^{2}}$$

where: Z = 1.96 (95% confidence level). p = 0.50 (assumed prevalence of adequate TB knowledge due to lack of prior local data). d = 0.05 (margin of error).

Substitution

$${n}_{0}=\frac{\left(1.96{)}^{2}\times 0.50(1-0.50\right)}{\left(0.05{)}^{2}\right.}=\frac{3.8416\times 0.25}{0.0025}=\frac{0.9604}{0.0025}=384.16\approx 384$$

After accounting for non-response, the final sample size was set at 403 participants.

### Sampling procedure

A multistage sampling technique was employed. Selected districts in Mogadishu were randomly chosen, followed by random selection of neighbourhoods. Systematic random sampling was used to select households based on updated household listings. Within each selected household, one eligible adult respondent was chosen using simple random selection when more than one eligible individual was present.

### Data collection tool and procedure

Data were collected using a structured interviewer-administered questionnaire developed from established TB KAP survey tools and adapted to the Somali context. The instrument was prepared in English, translated into Somali, and back-translated to ensure consistency and semantic equivalence.

Data were collected electronically using KoBoToolbox to enhance accuracy and data security. A pre-test was conducted on approximately 5% of the sample in a non-selected area to assess clarity, relevance, and logical flow. Necessary revisions were made prior to the main data collection. Data collectors received training on study objectives, ethical considerations, and standardized interviewing techniques.

### Data management and statistical analysis

Data were exported from KoBoToolbox, checked for completeness and consistency, and cleaned before analysis using Stata version 17.

Descriptive statistics (frequencies, percentages, means, and standard deviations) were used to summarize sociodemographic characteristics and KAP responses.

Independent sample t-tests and one-way analysis of variance (ANOVA) were conducted to compare mean knowledge, attitude, and practice scores across sociodemographic categories. Statistical significance was determined at *p* < 0.05.

Internal consistency of the KAP scales was assessed using Cronbach’s alpha coefficients.

### Result of the study

Table1: Among the 403 study participants, the majority were aged 30–40 years (36.7%), followed by 18–25 years (32.3%), indicating that most respondents were young to middle-aged adults. Males constituted 60.5% of the sample, while females accounted for 39.5%. Over half of participants were married (51.6%), with 36.7% single and smaller proportions divorced (8.7%) or widowed (3.0%). A substantial proportion had no formal education (43.2%), although 37.7% had attained college or university education, reflecting marked educational variation within the population. Regarding household size, more than half of respondents lived in households with eight or more members (54.8%), suggesting relatively large family structures that may have implications for tuberculosis transmission dynamics.

**Table 1:** Socio-demographic characteristics of study participants (n = 403).

Variable	Category	Frequency (n)	Percent (%)
Age (years)	18–25	130	32.3
	25–30	83	20.6
	30–40	148	36.7
	40 and above	42	10.4
Sex	Female	159	39.5
	Male	244	60.5
Marital status	Single	148	36.7
	Married	208	51.6
	Divorced	35	8.7
	Widowed	12	3.0
Level of education	No formal education	174	43.2
	Primary	36	8.9
	Secondary	41	10.2
	College/University	152	37.7
Household size	2–3	77	19.1
	4–7	105	26.0
	8–10	149	36.9
	11 and above	72	17.9

Table 2: The majority of participants (85.4%) had heard of tuberculosis, indicating high general awareness of the disease. Most respondents correctly identified transmission through coughs or sneezes (87.1%) and recognized persistent cough as a common symptom (86.4%). Additionally, 83.1% were aware that TB is curable if diagnosed early. However, only 48.6% correctly knew that TB is caused by germs or bacteria, revealing a substantial gap in understanding the underlying cause of the disease despite relatively good awareness of its transmission and symptoms.

**Table 2:** Knowledge of tuberculosis among study participants (n = 403).

Variable	Response	Frequency (n)	Percent (%)
Ever heard of TB	Yes	344	85.4
	No	59	14.6
TB is caused by germs/bacteria	Yes	196	48.6
	No	207	51.4
TB can be transmitted through coughs/sneezes	Yes	351	87.1
	No	52	12.9
Persistent cough is a common symptom of TB	Yes	348	86.4
	No	55	13.6
TB is curable if diagnosed early	Yes	335	83.1
	No	68	16.9

Table 3: Most participants demonstrated positive attitudes toward tuberculosis. A large majority perceived TB as a serious disease (88.6%), supported early medical care seeking (83.6%), and would encourage someone with TB to visit a health facility (86.4%). Similarly, 83.1% believed in completing the full course of TB treatment. However, only 50.6% reported feeling comfortable being with someone who has TB, indicating the presence of stigma despite generally favorable attitudes toward treatment and care.

**Table 3:** Attitudes toward tuberculosis among study participants (n = 403).

Variable	Response	Frequency (n)	Percent (%)
TB is a serious disease	Yes	357	88.6
	No	46	11.4
People should seek medical care early for TB	Yes	337	83.6
	No	66	16.4
Would encourage someone with TB to visit a health facility	Yes	348	86.4
	No	55	13.6
Feel comfortable being with someone who has TB	Yes	204	50.6
	No	199	49.4
Believe in completing the full course of TB treatment	Yes	335	83.1
	No	68	16.9

Table 4: The overall KAP scores indicate moderate to good levels among participants. The mean knowledge score was 3.91 ± 1.23, attitude 3.92 ± 1.28, and practice 3.92 ± 1.15, on a scale of 0–5. Internal consistency was acceptable for knowledge (α = 0.64) and attitude (α = 0.68), but lower for practice (α = 0.47), suggesting that the practice items were less consistent in measuring a single construct.

**Table 4:** Knowledge, attitude, and practice scores toward tuberculosis (N = 403).

Variable	Mean ± SD	Range	Cronbach’s α
Knowledge	3.91 ± 1.23	0–5	0.64
Attitude	3.92 ± 1.28	0–5	0.68
Practice	3.92 ± 1.15	0–5	0.47

Table 5: Comparison of knowledge, attitude, and practice scores across sociodemographic variables with statistical significance.

**Table 5:** Comparison of mean knowledge, attitude, and practice (KAP) scores across sociodemographic characteristics (N = 403).

Variable	Category	Knowledge (Mean ± SD)	*p* value	Attitude (Mean ± SD)	*p* value	Practice (Mean ± SD)	*p* value
Sex	Female	4.06 ± 1.06	0.047	4.00 ± 1.04	0.330	4.16 ± 1.07	0.0009
	Male	3.81 ± 1.33		3.87 ± 1.41		3.77 ± 1.17	
Age	18–25	3.73 ± 1.69	0.046	3.60 ± 1.66	< 0.001	3.81 ± 1.26	0.0018
	25–30	3.88 ± 1.19		3.75 ± 1.09		3.63 ± 1.36	
	30–40	4.12 ± 0.75		4.32 ± 0.85		4.19 ± 0.79	
	≥ 40	3.74 ± 0.91		3.88 ± 1.17		3.93 ± 1.24	
Marital status	Single	3.71 ± 1.58	0.011	3.63 ± 1.61	0.0009	3.68 ± 1.29	< 0.001
	Married	4.10 ± 0.76		4.16 ± 0.79		4.22 ± 0.87	
	Divorced	3.77 ± 1.33		3.91 ± 1.50		3.51 ± 1.29	
	Widowed	3.42 ± 2.11		3.50 ± 1.93		3.08 ± 1.62	
Education	No formal	3.93 ± 0.84	0.565	4.06 ± 0.94	0.249	4.16 ± 0.92	0.0011
	Primary	3.67 ± 1.33		3.81 ± 1.55		3.50 ± 1.08	
	Secondary	3.80 ± 1.35		3.98 ± 1.13		3.61 ± 1.43	
	College/University	3.97 ± 1.53		3.78 ± 1.55		3.84 ± 1.26	
Household size	2–3	3.40 ± 1.89	0.0007	3.34 ± 1.83	< 0.001	3.66 ± 1.41	0.0124
	4–7	3.93 ± 0.91		4.28 ± 0.91		3.97 ± 0.99	
	8–10	4.07 ± 0.88		4.01 ± 1.06		4.12 ± 1.05	
	≥ 11	4.07 ± 1.27		3.86 ± 1.25		3.72 ± 1.19	

The Table 5 shows comparison of KAP scores across sociodemographic groups reveals important patterns. Females had significantly higher knowledge (4.06 vs. 3.81; *p* = 0.047) and practice scores (4.16 vs. 3.77; *p* = 0.0009) than males, although attitude scores were similar. Age was strongly associated with KAP: participants aged 30–40 years had the highest knowledge (4.12), attitude (4.32), and practice (4.19) scores, while younger adults (18–25) scored lowest, indicating that experience and maturity may enhance TB-related understanding and behaviours. Married respondents had significantly higher scores across all domains compared to single, divorced, or widowed participants, suggesting household responsibilities influence awareness and practice. Education was significantly associated only with practice (*p* = 0.0011), with no formal education surprisingly showing high practice scores, possibly reflecting practical experience or exposure to health messaging outside formal schooling. Household size influenced all KAP domains, with mid-sized households (4–10 members) generally scoring higher than smaller (2–3 members) or very large households (≥ 11 members), highlighting the role of social exposure and household dynamics in shaping TB knowledge, attitudes, and practices.

## Discussion

The principal findings of this study reveal a significant epidemiological paradox: while general awareness of human tuberculosis (TB) is nearly universal among households, specific biomedical knowledge regarding its etiology, transmission, and prevention remains dangerously deficient. In Mogadishu, although over 90% of household heads recognized human TB, only a small fraction identified its biological cause, with many attributing the disease to traditional concepts such as “cold toxins” or “bad air”^1^. This findings-practice gap is further evidenced by the fact that despite high awareness, only a third of expected annual cases are detected and treated^1^. This disconnect between “hearing” of TB and “understanding” it mirrors patterns seen in rural Chad, where 90.6% of participants showed good knowledge but only 28.7% exhibited good practices^11^. The most concerning pattern revealed is the significant ignorance regarding zoonotic transmission, as the vast majority of households in Mogadishu were unaware of animal TB or the risks of bovine TB^1^. This indicates a critical failure in community health literacy that allows for ongoing, unrecognized transmission routes within the domestic environment.

Interpreting these findings in the context of global and regional evidence highlights both unique local challenges and common behavioral themes across high-burden settings. The reliance on non-biomedical explanations for TB in this study aligns with data from rural southwest Ethiopia, where 50.4% of TB suspects attributed the illness to the “evil eye”^22^. Similarly, the tendency to confuse TB with the common cold or minor ailments is a recurring theme in Malawi and Indonesia, often leading to a "wait and see" approach that delays diagnosis for weeks or even years^17,26^. However, the study setting in Somalia presents intensified barriers compared to more stable regions. Unlike in South Africa or India, where free treatment awareness is higher, the collapse of health infrastructure during conflict has created a deep-seated reliance on informal providers and family advice^1,5,15^. The observed discordance in transmission knowledge where households might correctly identify airborne routes yet still erroneously prioritize avoiding shared utensils is consistent with findings in The Gambia^10^. Such inconsistencies suggest that while public health messaging has introduced the concept of respiratory spread, it has not yet successfully dismantled deeply ingrained cultural myths regarding contagion.

Household knowledge gaps and cultural misconceptions serve as primary drivers of delayed diagnosis and suboptimal treatment outcomes. When TB is perceived not as a bacterial infection but as a “shameful” disease linked to “sinful behavior” or promiscuity, as seen in Malawian and Vietnamese studies, the immediate reaction is concealment rather than care-seeking^12,17,27^. This study’s finding that households often wait for informal treatments to fail before visiting a clinic is a critical barrier to the "End TB Strategy^5,22^. In fragile settings, the belief that TB is a “death penalty” or incurable leads to psychological distress and social withdrawal^28^. Furthermore, the misconception that TB is purely hereditary a belief identified in both Bangladesh and Malawi detracts from the urgency of contact tracing, as family members may view the disease as an inevitable genetic fate rather than a preventable infection^17,20^. These attitudes create a “no talk” environment, where the fear of being “gossiped about” or “mocked” outweighs the perceived benefit of early medical intervention^17^.

Structural and contextual determinants, particularly the intersection of poverty and health system fragility, further complicate TB control. The study highlights that socioeconomic vulnerability, characterized by overcrowding and low educational attainment, is the most consistent predictor of poor knowledge and practice^16,24^. In urban centers like Mogadishu, high-density living in "mud or mixed houses" facilitates rapid droplet transmission, yet the cost of transportation and the fear of losing daily income prevent the poor from accessing centralized diagnostic services^17,22,29^. This is consistent with findings in Pakistan and Ethiopia, where the wealth index was directly proportional to TB knowledge and the ability to seek rapid treatment^16,29^. Gender dynamics also play a pivotal role; in many Sub-Saharan African contexts, women are less empowered in health decision-making and face higher economic vulnerability if they lose their capacity to work, making them more likely to face severe social isolation^9,17^. The additional layer of conflict-related displacement in Somalia intensifies these vulnerabilities, as displaced households lack the stable social networks that often facilitate information sharing in settled communities^11,12^. The implications for public health policy and practice are profound, necessitating a shift toward community-based, culturally tailored interventions. The findings underscore that "pure knowledge transfer" is insufficient; instead, there is an urgent need for social mobilization and practical training^11^. Utilizing mass media, particularly radio, remains the most effective way to reach both rural and urban households, as evidenced by its role as a primary information source in Chad and Somalia^1,10,11^. Education programs must move beyond general awareness to address specific misconceptions about the biological cause of TB and the safety of the domestic environment. Crucially, a “One Health” approach must be integrated into national TB control strategies to educate households on the zoonotic risks of consuming untreated animal products, bridging the current gap between human and animal health literacy^1^. Furthermore, engaging community health workers (CHWs) and traditional leaders to deliver messages in local languages can help destigmatize the disease by emphasizing that TB is curable and that everyone, regardless of their "morality," is susceptible to airborne infection^11,17^. This study has several strengths and limitations that must be considered when interpreting the results. A primary strength is the use of household-level data in a fragile, high-burden setting, providing a baseline for national policy that is often missing from larger global datasets^1^. However, the cross-sectional design precludes the establishment of causal relationships between demographic factors and health behaviors^6,21,23^. The reliance on self-reported data introduces potential social desirability bias, where respondents may overestimate their positive health practices to align with perceived expectations^12,15,16^. Additionally, the study was conducted in a specific urban district, which may limit the generalizability of findings to pastoralist or more remote populations who face different environmental risks and barriers to care^16,17^. Despite these constraints, the analytical rigor of the study provides a vital “signal” for health authorities to prioritize behavioral change over simple awareness^7^. In conclusion, this study demonstrates that while the population is aware of the existence of TB, deep-seated misconceptions and structural barriers continue to fuel a cycle of delayed diagnosis and community transmission. To move toward TB elimination in fragile settings, public health initiatives must prioritize stigma reduction and the correction of traditional myths through community-led health education^3,11,17^. Future research should utilize qualitative and longitudinal methods to explore the underlying emotional and sociocultural dynamics that drive the "intention to conceal" TB status within the family^9,12^. Intervention-based research is also needed to evaluate the effectiveness of integrating TB screening into routine primary care and utilizing digital tools to monitor treatment adherence in displaced populations^25,30^. Ultimately, strengthening the National TB Control Programme requires a multifaceted strategy that empowers households as active participants in the reporting and prevention chain, rather than passive recipients of medical services^11^.

## Conclusion

This study reveals that households in Mogadishu have moderate to good awareness of tuberculosis, particularly regarding its transmission and symptoms, but significant gaps remain in understanding its biological cause and zoonotic risks. Positive attitudes toward early care-seeking and treatment completion coexist with persistent stigma, as many respondents reported discomfort interacting with TB patients. Knowledge, attitudes, and practices were influenced by sociodemographic factors such as age, sex, marital status, education, and household size, highlighting the role of experience, responsibility, and social context in shaping TB-related behaviors. These findings underscore the need for culturally tailored, community-based interventions that address misconceptions, reduce stigma, and promote early diagnosis and adherence, alongside strategies that strengthen household engagement within national TB control efforts.

### Recommendations

- Early case detection: Promote recognition of key symptoms and timely care-seeking; implement household-based screening.
- Mass media engagement: Use radio and local channels to reach urban and peri-urban households.
- Gender- and age-sensitive strategies: Focus on youth and leverage women/married adults’ household decision roles.
- Monitoring and research: Conduct longitudinal/qualitative studies and evaluate interventions like digital adherence tools.
- Household involvement: Train households to report suspected cases and support treatment adherence.

## Data Availability

The datasets generated and/or analysed during the current study are not publicly available because the ethical approval obtained for this study does not permit public sharing of participant-level data. However, the datasets are available from the corresponding author on reasonable request.

## Abbreviations

ANOVA: Analysis of variance
CHWs: Community health workers
HIV: Human immunodeficiency virus
IRB: Institutional review board
KAP: Knowledge, attitude, and practice
LMICs: Low- and middle-income countries
MDR-TB: Multidrug-resistant tuberculosis
NTCP: National tuberculosis control programme
SD: Standard deviation
SSA: Sub-Saharan Africa
TB: Tuberculosis
